# A Review on Ion-Exchange Membrane Fouling during the Electrodialysis Process in the Food Industry, Part 1: Types, Effects, Characterization Methods, Fouling Mechanisms and Interactions

**DOI:** 10.3390/membranes11100789

**Published:** 2021-10-16

**Authors:** Lasâad Dammak, Julie Fouilloux, Myriam Bdiri, Christian Larchet, Estelle Renard, Lassaad Baklouti, Veronika Sarapulova, Anton Kozmai, Natalia Pismenskaya

**Affiliations:** 1 Institut de Chimie et des Matériaux Paris-Est (ICMPE), Université Paris-Est Créteil, CNRS, ICMPE, UMR 7182, 2 Rue Henri Dunant, 94320 Thiais, France; julie.fouilloux@u-pec.fr (J.F.); myriam.bdiri@u-pec.fr (M.B.); larchet@u-pec.fr (C.L.); e.renard@u-pec.fr (E.R.); 2 Department of Chemistry, College of Sciences and Arts at Al Rass, Qassim University, Ar Rass 51921, Saudi Arabia; bakloutilassaad@yahoo.fr; 3 Department of Physical Chemistry, Kuban State University, 149, Stavropol’skaya Str., 350040 Krasnodar, Russia; vsarapulova@gmail.com (V.S.); kozmay@yandex.ru (A.K.); n_pismen@mail.ru (N.P.)

**Keywords:** ion-exchange membrane, electrodialysis, food industry, foulant identification, fouling mechanisms

## Abstract

Electrodialysis (ED) was first established for water desalination and is still highly recommended in this field for its high water recovery, long lifetime and acceptable electricity consumption. Today, thanks to technological progress in ED processes and the emergence of new ion-exchange membranes (IEMs), ED has been extended to many other applications in the food industry. This expansion of uses has also generated several problems such as IEMs’ lifetime limitation due to different ageing phenomena (because of organic and/or mineral compounds). The current commercial IEMs show excellent performance in ED processes; however, organic foulants such as proteins, surfactants, polyphenols or other natural organic matters can adhere on their surface (especially when using anion-exchange membranes: AEMs) forming a colloid layer or can infiltrate the membrane matrix, which leads to the increase in electrical resistance, resulting in higher energy consumption, lower water recovery, loss of membrane permselectivity and current efficiency as well as lifetime limitation. If these aspects are not sufficiently controlled and mastered, the use and the efficiency of ED processes will be limited since, it will no longer be competitive or profitable compared to other separation methods. In this work we reviewed a significant amount of recent scientific publications, research and reviews studying the phenomena of IEM fouling during the ED process in food industry with a special focus on the last decade. We first classified the different types of fouling according to the most commonly used classifications. Then, the fouling effects, the characterization methods and techniques as well as the different fouling mechanisms and interactions as well as their influence on IEM matrix and fixed groups were presented, analyzed, discussed and illustrated.

## 1. Introduction

Electrodialysis (ED) was first established for water treatment applications, principal-ly for water desalination [1,2,3,4] and is still highly used and recommended in this field for its high water recovery, long lifetime compared to other usual technologies and acceptable electricity consumption [5]. In addition to that, the ED process is very flexible and is basi-cally regulated to be an efficient desalination method that could be controlled by fixing the current (or potential drop) in the process. Today, thanks to technological progress related to the development of ED processes and the emergence of new ion-exchange membranes (IEMs) [6,7,8,9,10], this technique has been extended to other applications, for example, in the food industry for milk demineralization, deacidification and demineralization of bever-ages such as sugarcane and cranberries juices, tartaric stabilization of wine, desalination of cheese whey, treatment of glucose syrup, [11,12,13,14,15] etc.

This expansion of uses has increased the interest in ED techniques but has also gen-erated several problems such as limitation of IEM membrane lifetime due to different age-ing mechanisms (prolonged use, cleaning operations, fouling phenomena, etc.) [16,17,18]. If these aspects are not sufficiently controlled and mastered, the use and the efficiency of ED processes will be limited, since it will no longer be competitive or profitable compared to other treatments and separation methods.

In our present work, we focused on the issues of IEM fouling in the ED industry and it should be noted that this is a subject of common interest to all types of membranes used in all baromembrane [2,19,20] and electromembrane [21,22,23,24] methods. As was defined and explained in our previous review on IEM cleanings and strategies of fouling prevention during ED in the food industry [25], the terms “fouling” and “scaling” designate organic matters and mineral matters, respectively, and the same definition has been adopted in this review.

Commercial IEMs show excellent performance in the ED processes, especially with new progress in membrane synthesis and manufacturing. However, the organic foulants such as proteins, surfactants, polyphenols or other natural organic matters [26,27,28,29,30] can adhere to the membrane surface forming a colloid layer or can infiltrate the membrane matrix, which leads to the increase in electrical resistance, resulting in higher energy con-sumption, lower water recovery and lifetime limitation [22,24,31,32]. Until now, mem-brane fouling has still represented a severe problem in the ED processes, especially when using anion exchange membranes (AEMs) [33,34]. IEM fouling not only can increase the electrical resistance but also decreases the lifetime of these membranes [5,35].

**Figure 1:**
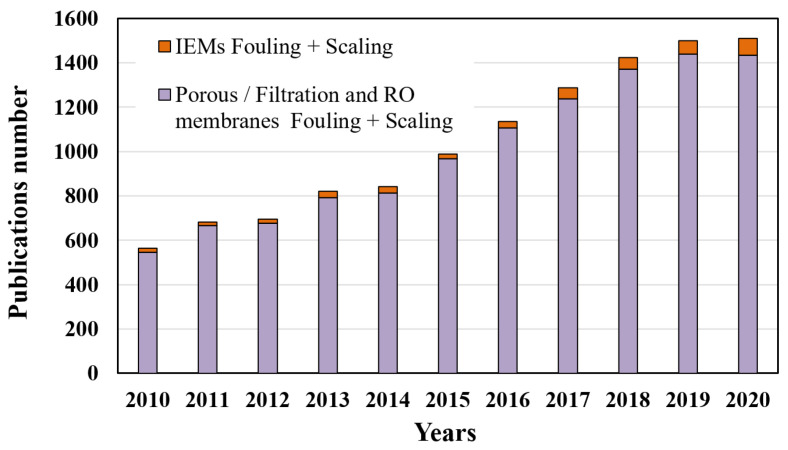
Evolution of the number of publications on the topic of fouling and scaling of ion-exchange membranes vs. other industrial filtration membranes from 2010 to 2020. Source of statistics: Web of Science. Keywords: “ion-exchange membrane” AND “fouling” OR “ion-exchange membrane” AND “scaling” OR “porous membrane” AND “fouling” OR “porous membrane” AND “scaling” OR “filtration membrane” AND “fouling” OR “filtration membrane” AND “scaling” OR “reverse osmosis membrane” AND “fouling” OR “reverse osmosis membrane” AND “scaling”.

The interest in fouling, scaling and ageing phenomena has generated an increase in the number of studies and publications that deal with the different aspects of these issues. In this context, we propose to present the results of a statistical study on the evolution of the amount of published scientific work and research carried out on the themes of fouling and/or scaling of different membrane types during the last decade. Figure 1 presents the evolution of the number of publications on the topic of fouling and scaling of IEMs com-paring to other industrial filtration membranes from 2010 to 2020.

A continuous increase in the number of publications can be seen for both electromembrane and baromembrane technologies during the last decade, which confirms the growing interest for membrane fouling phenomena. It should also be noted that studies on IEMs do not exceed 5–6% of the published works on these themes each year. The slowdown in the rate of increase in the number of publications in 2020 is probably related to a decrease in scientific activity following the global health crisis. The large number of publications on porous, filtration and reverse osmosis (RO) membranes is due on the one hand to the older development of these techniques, and on the other hand to a greater diversity of their applications.

**Figure 2:**
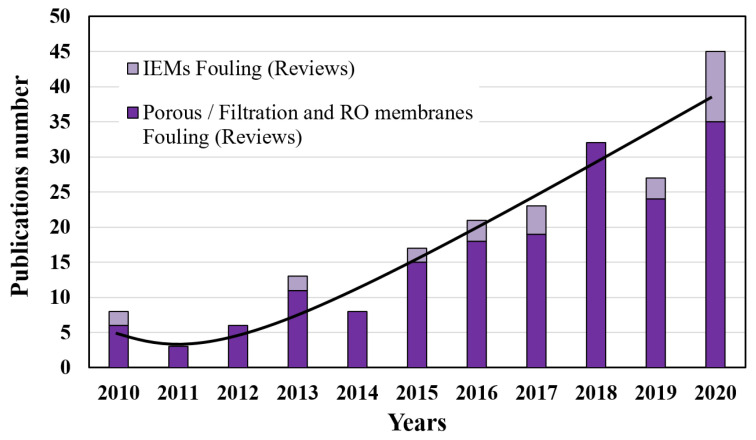
Evolution of the reviews number on the topic of ion-exchange membrane fouling vs. other industrial filtration membranes from 2010 to 2020. Source of statistic: Web of Science.

Figure 2 presents the evolution of the number of reviews on the topic of IEM fouling comparing to other industrial filtration membranes from 2010 to 2020. An overall increase in the number of reviews can be observed over the last 10 years despite some fluctuations from year to year. The ratio of the works carried out on the IEMs to that carried out on the filtration membranes varies between 0% and 22% in the last decade. In all cases, the number of reviews on IEM fouling is always lower or even negligible. Therefore, it is essential to further develop the studies and the research carried out on the fouling phenomena of IEMs.

The present work reviewed a significant amount of scientific publications, research and reviews studying the phenomena of IEM fouling during ED process in food industry with a special focus on the last decade. It first classified the different types of fouling ac-cording to the most commonly used classifications. Then, the fouling effects, the charac-terization methods and techniques and the different fouling mechanisms and interactions are presented and illustrated.

## 2. The Nature of Foulants and Methods of Their Identification

The word “fouling” was used as a generic term to talk about all types of fouling in-cluding organic, inorganic, biological and colloidal matters in old publication [21,36]. Grossman et al. [21] used the term “fouling” to refer to the formation of a layer of impuri-ties on the surface of the ion exchange membrane (IEM) and “poisoning” to refer to their penetration inside the membrane. In some cases, there is a confusion between the terms “clogging” and “fouling”. The latter term can be used for both IEMs and filtration mem-branes [37] but the term “clogging” is more appropriate for porous membranes when talking about cake formation on the filtration membrane surface due to pore clogging and pore blocking or clogging of membrane defects by a layer of foulants [38,39]. 

It would be more judicious to use a common vocabulary to designate these phenom-ena for the community that works on these issues. In this review as in some recent studies, the word “fouling” refers to organic matters specifically but can be used to designate all fouling phenomena independently of the foulants’ nature; the word “scaling” refers to mineral compounds and the terms “external” and “internal” designate the fouling and scaling of the membrane surface or bulk of the matrix, respectively. 

The fouling phenomenon is defined as the deposition of undesirable molecules or substances of different nature (particles, colloids, macromolecules or salts) on the surface of the membrane or their adsorption inside the matrix due to different interactions and mechanisms depending on the nature of the foulants and the membrane material. All foulants are classified by their size [40], nature [41], as well as by the strength of binding to the membrane material [41]. Substances that can enter liquid media of the food industry differ in size as follows [42]:

- Dissolved substances (size less than 1 nm), including ions of inorganic substances, as well as ions and molecules of organic acids, saccharides, amino acids, proteins, phe-nolic compounds, etc.
- Colloidal particles (size from 1 nm to 1 μm) formed by inorganic, organic substances or their mixtures, the surface of which has a positive or negative charge;
- Suspended particles (larger than 1 μm) include biological objects (viruses, bacteria, and fungi), fragments of biological cells, colloidal aggregates and salt crystals.

Classification of substances by their nature includes [41,43,44,45]: inorganic (mineral) substances; organic compounds; colloidal particles; biological objects. Such division is rather conditional, since some organic dissolved compounds under certain conditions become colloidal particles, and not only salts of inorganic (gypsum, CaSO_4_ 2H_2_O), but also those of organic (potassium hydrogen tartrate, KHT) acids can be poorly soluble [46].

In recent years, great strides have been made in the identification of foulants and in the study of their morphology in systems with IEMs. Some of them are examined below.

### 2.1. Identification of Foulants

#### 2.1.1. Mineral Foulants

**Figure 3:**
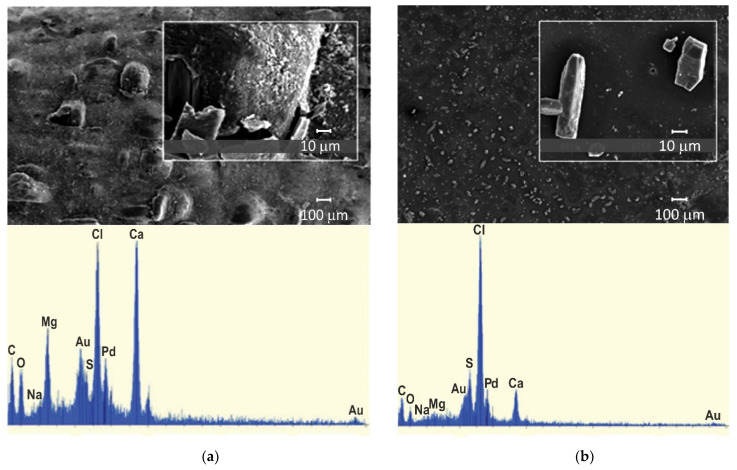
SEM images and EDS spectrograms of Neosepta CMX-Sb (Astom, Shunan, Japan) cation exchange membrane after operation in ED milk processing at pH = 5.4: surface facing to concentrate (**a**) or diluate (**b**). Adapted from [50].

**Figure 4:**
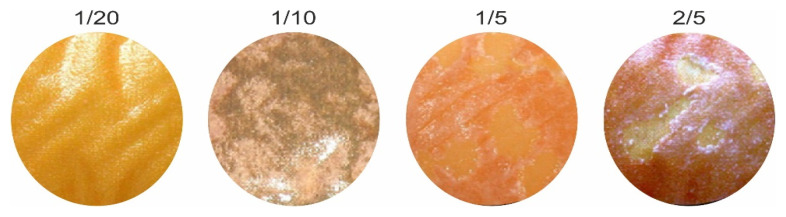
Photographs of Neosepta CMX-Sb membrane taken from the side of concentration compartment after ED treatment of solutions imitating whey (pH = 12). Numbers above the photo show magnesium/calcium ratios in the solution. Adapted from [58].

**Figure 5:**
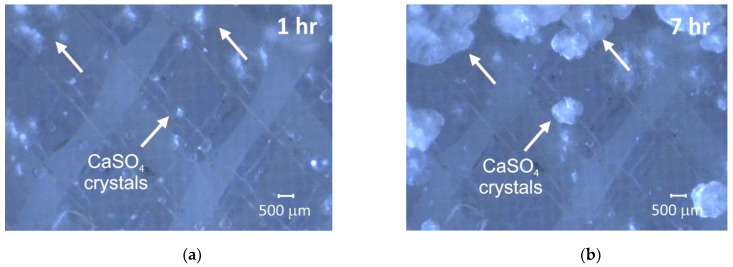
In-situ optic observation of scaled Selemion AMV (Asahi Glass, Tokyo, Japan) anion exchange membrane after 1 (**a**) and 7 (**b**) hour operation in a solution containing Ca^2+^ and SO_4_^2−^ ions. The study was carried out using a stereo microscope (Zeiss Discovery V8 Stereosope with total magnitude of X6.3–50.4, Norway) connected to a digital camera (Power-shot A640, Canon). Adapted from [48].

**Figure 6:**
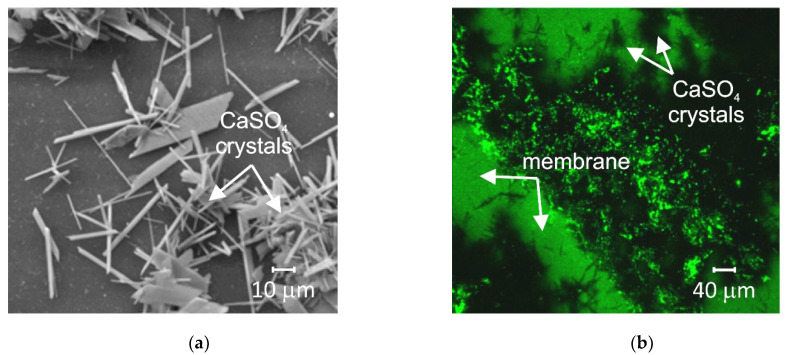
SEM (**a**) and 3D fluorescent optic (**b**) images of gypsum deposit on reverse osmosis RE182 (CSM Co., Seoul, Korea) membrane surface (**b**). Adapted from [52].

Mineral precipitation and crystal growth on the surface and in the pores of IEMs are characteristic of electrodialysis (ED) water conditioning or whey demineralization. Tradi-tionally, this type of fouling has been studied using scanning electron microscopy (SEM) combined with energy dispersive X-ray spectrometry (EDS) [47,48,49,50]. SEM allows one to determine the localization of mineral deposits and to identify the formed crystals by their shape. The EDS in turn gives an idea of the chemical composition of crystals and amor-phous formations. For example, gypsum crystals have a characteristic acicular structure, as shown in Figure 3. X-ray diffraction (XRD) or X-ray absorption fine structure (EXAFS) methods [47,51] also seem to be very informative for identifying crystallographic inorgan-ic foulants. It should be noted that drying of the samples precedes the use of these meth-ods. This preparation can lead to the destruction of membranes and crystals. In addition, the listed methods can only be applied when removing the IEM from the dialyzer, electro-dialyzer, or membrane reactor. Therefore, high-resolution optical microscopy is becoming more and more popular. This method allows one to study swollen membranes, as well as to determine the IEM color depending on the pH and the composition of the bathing solu-tion (Figure 4). Using optical microscopy, successful attempts have been made to study scaling in situ [48] (Figure 5). In recent years, antiscalants that have fluorescent properties have been developed. Embedding in the structure of gypsum crystals [52,53], these an-tiscalants are indicators of sediment on the membrane surface. Thus far, such studies have been carried out in relation to reverse osmosis (RO) membranes (Figure 6). However, this method can become very informative for studying scaling (fouling by mineral components) in IEM systems.

**Figure 7:**
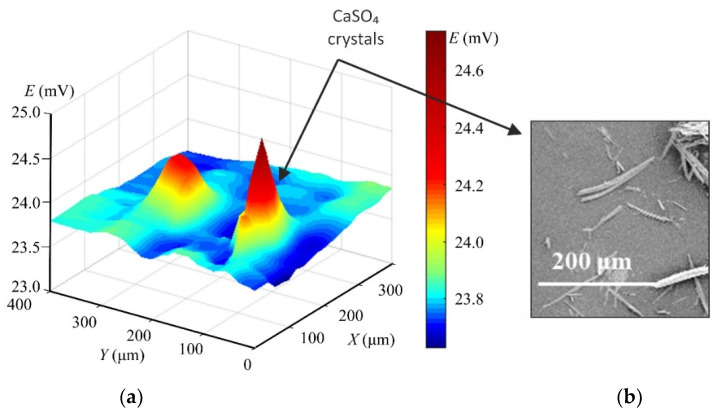
SECM 3D potential drop distribution at the surface of the swollen Neosepta CMX (Astom, Shunan, Japan) membrane with CaSO_4_ crystals (**a**) and SEM image of the same (dry membrane) surface (**b**). Adapted from [59].

The relatively new methods “Scanning ion conductance microscopy” (SICM) [54] and “Scanning electrochemical microscopy” (SECM) [55] allow scanning of the IEM sur-face and visualization of conductive and non-conductive areas that represent, within some distance, the surface morphology of the test material. For example, in one of the modifications of these methods [56,57], a pair of very fine microcapillaries (of about 5 µm in diameter) measures the potential drop on both sides of the membrane in an ED cell at a given current density. These capillaries can move in three mutually perpendicular direc-tions (X, Y, Z). The processing of chronopotentiogramms measured in situ at the nodes of 2D or 3D lattice under real electrical, chemical and hydraulic conditions of ED provide information on the IEM surface geometry. Moreover, this geometry is in good agreement with SEM images of dry (Figure 7) or swollen membranes obtained using optical micros-copy.

Comparison of the results of blank experiments carried out, for example, in a NaCl solution, with the data obtained in multicomponent solutions prone to precipitation, al-lows one to monitor the scaling process and its effect on the conductivity of the membrane surface [59].

#### 2.1.2. Organic Foulants and Colloidal Particles

Organic molecules of BSA, humate, carbonic acids, amino acids, anionic surfactants such as SDBS, peptides [13,24,34,46,60,61,62,63,64,65,66,67,68,69] and more recently phenolic and polyphenolic compounds including anthocyanins [26,31,32,70,71,72,73,74,75] are the main substances that are found in the industrial environments of the food industry or their imitations. Many organic foulants are known to be negatively charged at neutral pH. The interaction of such substances with Ca^2+^ and Mg^2+^ cations and positively charged fixed groups is the reason for the formation of a thick gel-like film on the anion exchange membrane (AEM) surface [76,77,78]. At the same time, the presence of oily compounds in the processed solution does not lead to such fouling. The difficulty in identifying organic foulants lies in the fact that the processed solutions, as a rule, contain not one, but several substances close in elemental composition. For example, wine and fruit or berry juices can contain up to 600 components [26,31,32,79,80]. Moreover, the chemical structure of IEM and foulants is often too similar. Inside membranes, foulants can form compounds that were not present in the processed fluids [79,80].

##### Identification of Typical Chemical Elements

**Figure 8:**
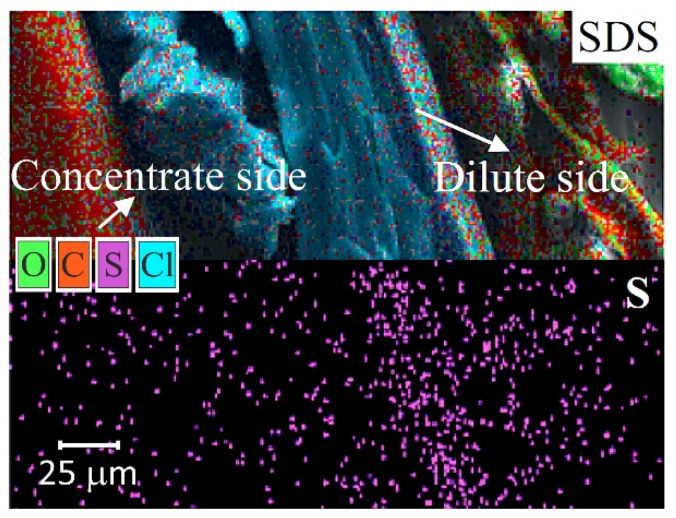
EDS element mapping of the fouled anion-exchange membrane cross-section. Foulant is sodium dodecyl sulfate (SDS). Adapted from [82].

The EDS method is widely used for the identification of organic foulants or other substances on the IEM surface [81,82] (Figure 8). At the same time, drying of IEM, preced-ing, for example, SEM and EDS, has a destructive effect on the colloidal particles and the three-dimensional structure of most organic foulants. In addition, EDS gives reliable re-sults only if the depth of the analyzed layer is more than 1 µm and is of little use for the analysis of thinner layers [83]. This limits the use of the traditional method of SEM-EDS and requires the employment of other suitable methods of identifying substances.

A wide range of methods are used to identify typical chemical elements characteristic of foulants. For example, Cheesman et al. [84] used ^31^P nuclear magnetic resonance spec-troscopy, molybdate colorimetry and inductively coupled plasma optical emission spec-trometry to identify phosphorus in organic and condensed inorganic compounds ad-sorbed by AEMs. X-ray photoelectron spectroscopy (XPS) or Rutherford backscattering spectroscopy (RBS) is used to analyze the elemental composition of a thin (up to 10 nm) surface layer [51,83]. Note that XPS and RBS are informative if the chemical composition of foulants differs significantly from that of the IEM [83,85,86]. However, they are not very useful for identifying proteins, amino acids, carboxylic acids and polyphenols, which are composed of the same chemical elements as the IEM.

**Figure 9:**
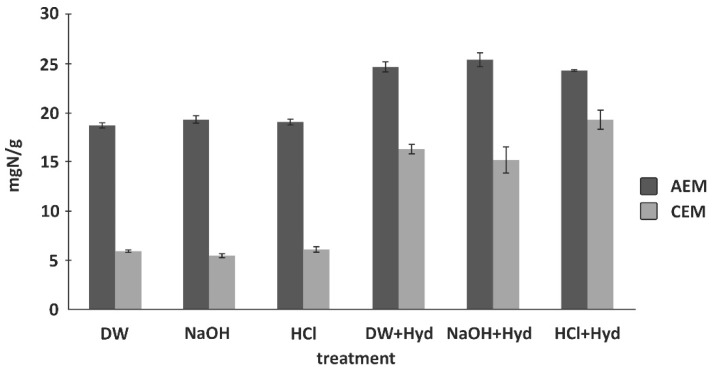
Evaluation of nitrogen content of CMX-Sb and AMX-Sb before (DW, NaOH, HCl) and after soaking in soy protein hydrolysate solution (DW + Hyd, NaOH + Hyd, HCl + Hyd) for 24 h (adapted from [63]). IEMs were pretreated in distilled water (DW), 1 M NaOH or HCl before the experiment.

Total nitrogen content analysis is mostly used for the detection and identification of proteins, peptides, amino acids, and other low molecular weight compounds [13,62,63,64,65,87] in IEM. The total nitrogen content can be determined using the Dumas method. In this method, dried membrane samples are combusted in the presence of O_2_ at high temperature of about 900 °C to 1000 °C to convert the sample into CO_2_, water and nitrogen. Then, the products of combustion are passed through a thermoelectric cooler to remove the water. The nitrogen gases are quantified using a thermal conductivity detector where He gas is used as reference. For the study of IEM fouling, several authors have used LECO nitrogen quantification method which is simple, rapid and automated but leads to mem-brane destruction [64]. This technique requires large sample size of about 150 mg dry membrane to obtain accurate and reproducible results. A pristine AEM, which contains fixed amino groups, is usually used as reference or control [79]. For example, Langevin et al. [63] used nitrogen content analysis to investigate the fouling of Neosepta CMX-Sb and AMX-Sb (Astom, Shunan, Japan) with a soy protein hydrolysate solution (SPHS) and study the effects of the different pre-treatments on fouling formation. The results showed that that cation exchange membrane (CEM) was almost two times more sensitive to peptide fouling than AEM. The nitrogen content observed for the CEM pretreated in HCl solution after soaking in the SPHS would be 12% higher than the ones pretreated by distilled water and NaOH (Figure 9). Persico et al. [65] preconized these methods to characterize peptide fouling on AEMs by a tryptic whey protein hydrolysate where it appeared that peptide charge modifications related to alkalinization, have a severe impact on AEM fouling. The authors also studied the formation of peptide layers and its adsorption mechanisms on AEMs and CEMs [63] depending on the pH of feed solution.

##### Identification of Characteristic Chemical Groups

Attenuated total reflection infrared spectroscopy (ATR–FTIR) is one of the most commonly used methods for the identification of organic fouling for IEMs and other membranes [56,57,59,67,88]. For example, Suwal et al. [80] used the ATR–FTIR to investigate the membrane surface (facing the anode or cathode) sensitive to peptide or amino acid fouling and deterioration after their use in consecutive ED-UF treatments. It should be noted that, in attenuated total reflection mode, this spectroscopy allows the identification of functional groups present on a thickness of about 1 µm.

**Figure 10:**
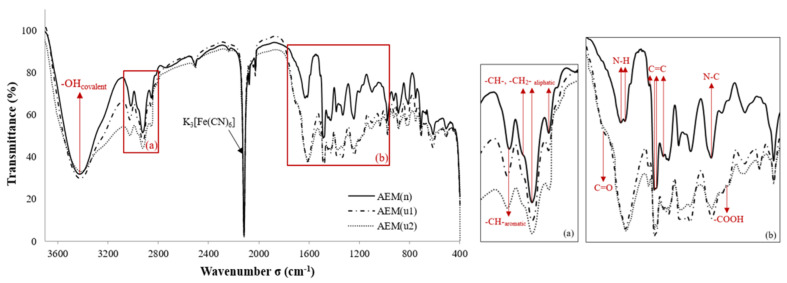
ATR–FTIR spectra of pristine (AEM(n)) and fouled (AEM(u1), AEM(u2)) anion exchange membrane Neosepta AMX-Sb used in the tartaric stabilization of red wine [31].

Bdiri et al. [26,31,32] investigated the fouling of Neosepta AMX-Sb and CMX-Sb used for the tartaric stabilization of red wine by phenolic compounds. The comparison and the interpretation of the ATR–FTIR spectra of pristine and fouled membranes (Figure 10) showed, principally, the presence of highly hydrated C=O, –COOH due to the accumulation of phenolic acids and phenolic compounds in AEMs which confirmed the affinity of organic acids for AEMs. The results also showed the intensification of C=C stretching bands of polyphenol aromatic rings in both membranes but this was more intense in CEMs.

**Figure 11:**
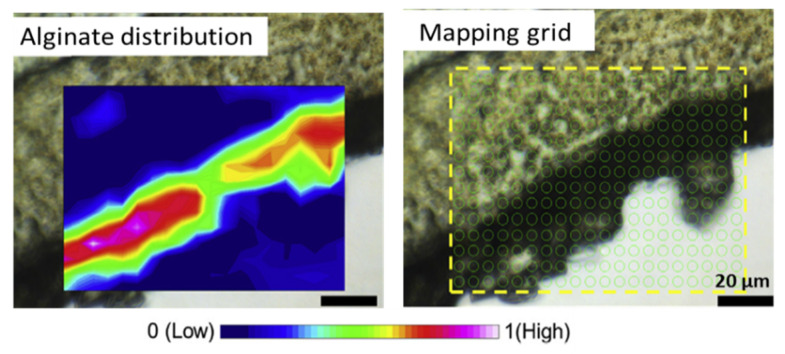
Synchrotron Fourier transform infrared mapping of alginate distribution and abundancy in the cross-section of membrane (in the left) and mapping grid (in the right). Spectrum integration of characteristic wavenumber was carried out to plot the map. Adapted from [89].

Xie et al. demonstrated the capabilities of the well-resolved Synchrotron Fourier transform infrared mapping [89] for quantifying organic foulants such as alginate (Figure 11) in membrane distillation. We believe that this relatively new method can be extremely useful for obtaining information on the localization and accumulation of specific organic foulants in the case of IEMs as well.

**Figure 12:**
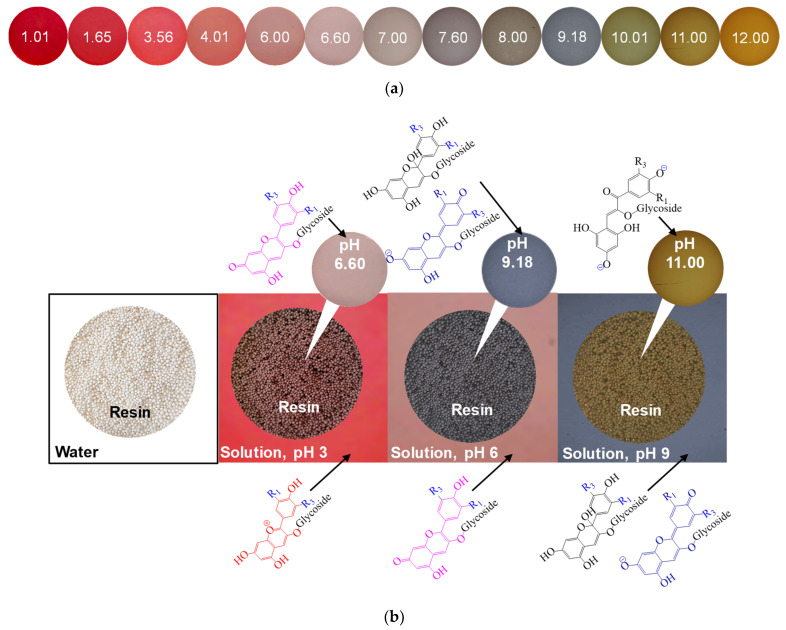
Colors of anthocyanin mixture solution depending on pH (**a**) as well as color of the aromatic anion-exchange resins AV-17-2P (KHIMIMPEX LLC, Kiev, Ukraine) equilibrated with distilled water and anthocyanin solutions of pH 3, 6 or 9 (**b**). Adapted from [92].

It is known that some organic groups are chromophores and fluorophores. These properties allow the use of classical optical microscopy and optical spectroscopy tech-niques to study the participation of these substances in fouling [90]. For example, the rep-resentatives of polyphenols—anthocyanins, are key substances in the fouling of ion ex-change materials used for wine stabilization and juice conditioning. These substances contain a chromophore group, the structure of which (and the color of anthocyanin) depends on the pH of the medium [91]: red flavylium cation (pH < 3); colorless carbinol pseudobase (pH = 4~5); purple quinoidal anhydrobase (pH = 6~7); deep blue anhydrobase anion (pH = 7~8); green anhydrobase dianion (pH = 8~10) and yellow chalcone dianion (pH > 11). In natural solutions containing a mixture of anthocyanins, the color palette may undergo some changes, but the general trend remains (Figure 12a). It was shown in [92] that this property of anthocyanins can be used to assess their structure within ion-exchange materials. Indeed, the color of aliphatic ion exchange resins equilibrated with anthocyanin-containing solutions (pH from 3 to 9) is in good agreement with the ATR–FTIR results. However, ATR–FTIR provides little information in the case of IEM, which has an aromatic matrix, due to the similarity of its structure to that of anthocyanins. In this case, changes in the color of the studied samples, for example aromatic anion exchange resin AV-17-2P (KHIMIMPEX LLC, Kiev, Ukraine), (Figure 12b) are easy to estimate using optical microscopy and the color indication scale [33,92]. Sarapulova et al. [33,92], using this method, established that the pH of the internal solution is shifted to acidic (cation exchange resins and CEMs) or alkaline values (anion exchange resins and AEMs) as compared to an equilibrium solution. Therefore, the structure of anthocyanins inside ion-exchange materials differs from their structure in an external solution (Figure 12b). The reason is Donnan exclusion of co-ions [93]: hydroxyl ions or protons, that are products of protonation–deprotonation of polar groups, substances entering the IEM or water.

**Figure 13:**
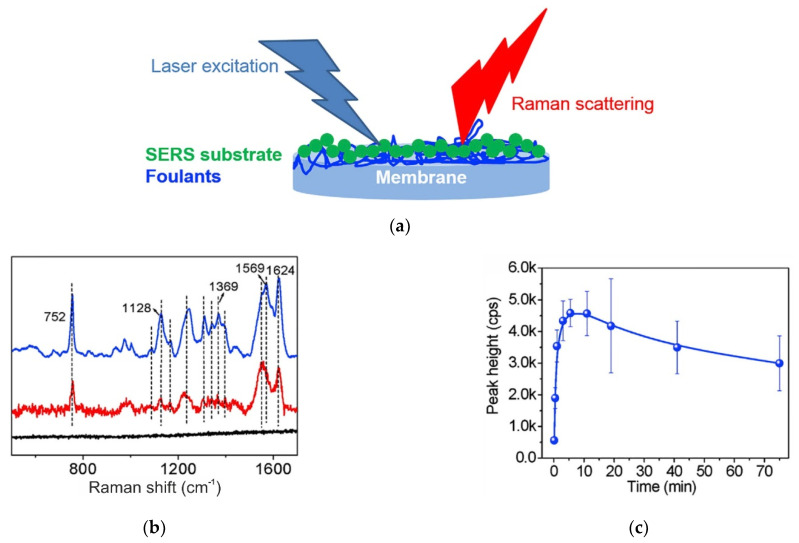
Surface-enhanced Raman Spectroscopy applied to monitor PVDF membranes fouling by proteins. (**a**) is a schematic illustration of SERS measurement; (**b**) is SERS spectrum of myoglobin on a PVDF membrane (blue line) with Ag sol, Raman spectrum of pure myoglobin solid (red line), and myoglobin on a PVDF membrane without Ag sol (black line). (**c**) is obtained at 752 cm^−1^. Adapted from [97].

Another alternative to ATR–FTIR is Raman spectroscopy, the use of which does not require preliminary drying of the studied membrane samples [51,94]. Surface-enhanced Raman spectroscopy (SERS) and Tip-enhanced Raman spectroscopy (TERS) can be used for real-time monitoring of membrane fouling. For example, Virtanen et al. [95] used Ra-man spectroscopy to study the interactions between the surface of the polyethersulfone membrane and vanillin, which is a foulant. Chen et al. [96] studied the adsorption and aggregation of proteins and polysaccharides in the pores of microfiltration membranes. Figure 13 illustrates an example of SERS application to study the adsorption of myoglobin on the surface of polyvinylidene fluoride (PVDF) membrane. Here, the peak height was determined as 752 cm^−1^. Note that this technique is limited by the high fluorescence of some polymers constituting the membranes, such as polystyrene-based membranes.

Fluorescence-based techniques have become increasingly popular in recent years. Fluorescence spectroscopy, the UV-vis spectra and fluorescence excitation–emission ma-trix (EEM) analysis allow detection of the UV humic-like substances’ adsorption on ion exchange resins [98]; EEM coupled with parallel factor analysis (PARAFAC) can provide some structural information about the metal binding with some organic matter, as well as organic substances (for example humic acid and protein) interactions [98,99,100,101,102]; two di-mensional fluorescence/Fourier transform infrared correlation spectroscopy gives infor-mation concerning the localization of functional groups of humic substances in complex metal cations [103]. For example, Peiris et al. [104] applied a fluorescence-based technique in combination with the Surface plasmon resonance (SPR) method to study the adsorption of α-lactalbumin, protein-like matter as well as colloidal particles. They found that inter-particle or inter-molecular physical-level interactions between these substances can be detected by signal attenuation in the SPR measurements.

##### Identification of Substances Included in the Composition of Foulant

Spectrophotometric techniques, where each of the components are determined at a given wavelength [92,105], are the simplest and most commonly used methods for the quantitative determination of various foulants in solutions. For example, the mass con-centration of the sum of anthocyanins in terms of cyanidin-3-glucoside is determined based on the change in the light absorption (wavelength of 510 nm) when the pH of the studied solutions changes from 1 to 4.4 units [92]. Protein concentration is measured at 595 nm using the chromogenic agent Coomassie brilliant [105], etc.

**Figure 14:**
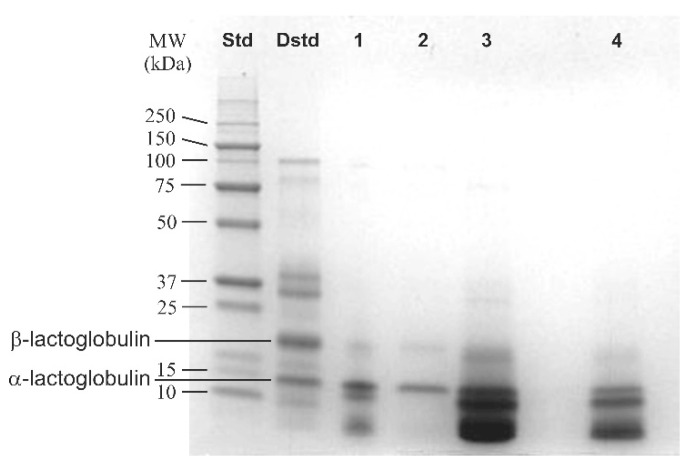
SDS-PAGE profile of molecular weight standard (Std), moleculer weight dairy standard (Dstd), whey protein hydrolysate before centrifugation (1), supernatant (2), precipitate (3) and gel on the AEM surface after electrodialysis (4). Adapted from [68].

Sodium dodecyl sulphate–polyacrylamide gel electrophoresis (SDS-PAGE) allows separation of proteins based on differences in their molecular weight (Figure 14) [68]. Mo-lecular weight (MW) standards are used for protein identification.

The Fourier transform-ion cyclotron resonance-mass spectrometry (FT-ICR-MS) method should also be mentioned. Ray et al. [106] are sure that FT-ICR-MS is the most in-formative tool to detect organic matters at molecular levels.

Chromatographic methods are widely used for IEM fouling investigation [26,46,68,72,73,105,107,108,109]. In the case of organic fouling with substances of different molecular weights, Size-exclusion (SEC) High-Liquid Performance Chromatography (HPLC) was found to be an effective method to identify foulants [46,107,108,109]. For exam-ple, Bdiri et al. [26] developed an extraction method of organic foulants from fouled Ne-osepta AMX-Sb and CMX-Sb with phenolic compounds during tartaric stabilization of red wine by ED. A mixture of four solvents at 25% volume each (acetone, methanol, isopropanol and ultra-pure water) was used. Then, specific Ultra-High-Liquid Performance Chromatography (UPLC) and HPLC methods were performed for the identification of phenolic compounds extracted and it was, for the first time, possible to identify numerous phenolic compounds adsorbed in the membranes (examples in Table 1).

Mass spectrometry (MS) coupled to the UPLC (UPLC-MS) [68] or to HPLC (HPLC-MS) [63] gives important and significant information about organic fouling and leads to a specific identification of the fouling nature by allowing choice of the appropriate chromato-graphic method to analyze each sample. Note that a necessary requirement for the application of this method is the preliminary extraction of foulants from the IEM by extraction methods that have to be previously optimized and have the appropriate standards. Aqueous solutions of mineral salts [110], individual organic substances [101,111] or mixtures of organic solvents [26,73] are used as stripping solutions. The samples are preliminarily subjected to finer grinding [73] to ensure the extraction of high molecular weight compounds, for example, proanthocyanidins, from the IEM. In the course of such pre-treatment, colloidal particles and some chemical compounds are destroyed; the extraction of foulants from the membrane may be incomplete, etc. The macromolecules of food polyphenols and anthocyanins [112,113], protein mixtures [114], etc., adsorbed onto the membrane surface are desorbed with a laser adsorbing solution known as a MALDI matrix and directly analyzed in MS [114].

#### 2.1.3. Biofouling

Membrane biofouling starts with the attachment of microbial cells at their surface. The interactions underlying this initial attachment are hydrophobic and electrostatic, leading to cell growth and multiplication thanks to soluble nutrients present in the feed water or organic foulants already present as a conditioning film. The microorganism me-tabolism will then excrete extracellular polymeric substances (EPS) which will create a three-dimensional matrix. EPS are high molecular weight substances being polysaccha-rides, proteins, nucleic acids and lipids, and account for 90% of biofilm dry mass [115]. In a nutshell, a biofilm is composed of microorganisms (10%w) living in a self-produced hydrated EPS matrix (90%w) providing mechanical stability, adhesion surface and an in-terconnected network promoting their metabolism.

**Figure 15:**
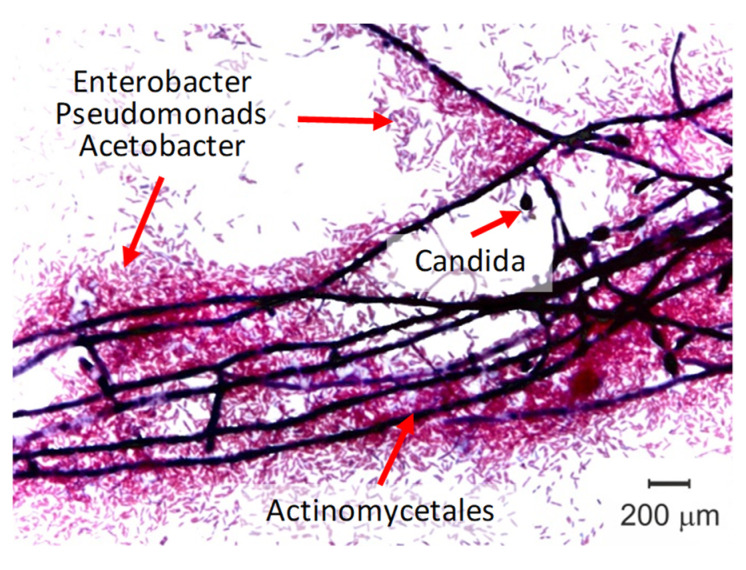
Optical images of the inoculation from touch smears taken from Neosepta AMX-Sb surfaces facing concentration compartment. Data obtained in a laboratory electrodialysis cell at tartrate stabilization of wine. Adapted from [67].

Biofouling is most often identified by standard microbiological methods [66]. In par-ticular, smear-prints of the membrane surface are applied to degreased glass slides, dried, and then stained by the Gram method using a carbolic solution of gentian violet, Lugol’s and fuchsin solution. Gram-negative microorganisms painted in a pink–red color, and Gram-positive microorganisms becomes blue–violet (Figure 15). Using this method, for example, it was found [67] that the side of AEM, which is exposed to the NaCl solution, is more susceptible to biofouling. The opposite side, which is in contact with wine and con-tains ethyl alcohol and polyphenols (that possess an antibacterial activity), hardly undergoes biofouling.

**Figure 16:**
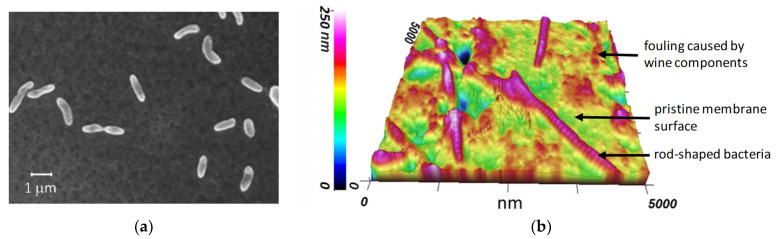
Detection of some microorganisms using (**a**) the emission scanning electron microscope (FE-SEM; image of Neosepta AMX (Astom, Shunan, Japan) membrane after 20 h contact with P. putida bacterial suspension. Adapted from [117]) and (**b**) the atomic force microscopy (AFM image of Neosepta AMX-Sb sample (air-dried) which was soaked in red wine during 72 h. Adapted from [33]).

Some of the microorganisms can be detected using SEM [116,117] (Figure 16a) or atomic force microscopy (AFM) [33,116] (Figure 16b).

The major disadvantage of SEM imaging is that samples are subjected to harsh treatments before visualization which can damage the biofilm. To overcome this issue, Environmental Scanning Electron Microscopy (ESEM), which is a form of SEM allowing imaging of hydrated specimens, can be used. This technique allows direct imaging of un-damaged hydrated biofilms at high magnification without prior preparation of the samples [118]. Biofilms are observed in their natural hydrated structure, thus avoiding any shrinkage compared to SEM. This technique has been used, for instance, by Luo et al. [119,120] to characterize IEM used in microbial desalination cells. It allowed them to highlight the formation of a porous biofouling gel-like layer on AEM surface, formed by bacterial growth. This visual observation was coupled and confirmed with EDS, which exhibited a high amount of carbon, oxygen, phosphate, sulfur, zinc, and potassium, as well as the absence of chlorine and fluorine present in AEM polymer. This confirms that the AEM surface was completely biofouled. Pictures are not as sharp as SEM ones, thus decreasing the optical analysis precision [121], but it is necessary when wanting to observe swelled/hydrated membranes. ESEM can be coupled with SEM [122] or AFM [123] to obtain higher resolution information.

In order to gain broader understanding of biofilm formation, identifying and quanti-fying microorganisms, as well as EPS, is crucial. As concerns over biofouling in ED pro-cesses are relatively new, few studies have been carried out to apply methods used in mi-crobiology for biofouled IEMs. However, those methods could be very useful to acquire a better understanding of biofouling, such as knowledge on deposition/adhesion mecha-nisms which could result in appropriate biofouling control strategies.

There are several techniques used to identify microorganisms, most of which start with DNA extraction followed by PCR amplification. In the case of 16S rRNA-gene se-quencing, DNA is then sequenced, and data are analyzed to allow determination of bacte-rial community diversity. The 16S rRNA gene is a commonly used genetic marker because of its sufficient length, presence in almost all bacteria and unaltered gene sequence over time. This technique is widely used to identify microorganisms in biofilm, for instance, of microbial desalination cells [120], as well as reverse osmosis membranes [123,124].

Quantitative real-time PCR (qPCR) is a fast method used to quantify the total number of microorganisms. Unknown DNA samples are compared against a standard curve of 16S rRNA genes [125] leading to an estimation of bacterial cell density [126]. Another way to evaluate bacterial cell density is Heterotrophic Plate Count (HPC) [125,127]. There is a wide variety of HPC methods, but their shared goal is to estimate the number of live and culturable bacteria in water.

After PCR amplification, DNA can also undergo a Denaturing Gradient Gel Electro-phoresis (DGGE), which is an electrophoresis technique using a chemical gradient to de-nature DNA moving across an acrylamide gel. This technique was used by Wu et al. [128] to identify microbial communities’ footprints attached on membrane bioreactors surfaces and their evolution with time. It allowed them to highlight that microbial communities shifted with solid retention time, therefore impacting EPS composition. The major ad-vantage of this technique is that it provides an easy and rapid estimation of microbial di-versity with less bias than traditional sequencing [129]; however, it should be noted that it is a destructive method. PCR-DGGE has also been used, coupled with 16S rRNA se-quencing, to characterize biofilm bacterial community composition in a nanofiltration membrane used for wastewater treatment [125,126] and also in microbial desalination cells for domestic wastewater treatment [120].

Terminal Restriction Fragment Length Polymorphism (T-RFLP) is another commu-nity fingerprint technique which uses PCR-amplificated DNA, with fluorescent tags, which is then digested by restriction enzymes leading to terminal restriction fragments of various lengths. Those fragments are then separated according to their size by capillary gel electrophoresis equipped with a fluorescence detector [130]. This method has been used by Chen et al. [131] and Gao et al. [127] to analyze the biofilm microbial community in membrane bioreactors (MBR).

Biofilms are diverse microbial assemblages and Automated Ribosomal Intergenic Spacer Analysis (ARISA) is a powerful technique for determining and estimating micro-bial richness and diversity. Vanysacker et al. [126] used ARISA to measure species rich-ness in a biofouled microfiltration membrane used in MBRs.

Fluorescence In situ Hybridization (FISH) is a molecular cytogenetic technique where fluorescent probes bind to chromosome parts with a high degree of complementarity. DNA samples first undergo dehydration, then staining with fluorescent probes, followed by observation using Confocal Laser Scanning Microscopy (CLSM) [132]. Genus-specific probes for FISH, such as rRNA-targeted oligonucleotide probes [125,132], have been de-veloped to enable specific and simultaneous identification of multiple microbial species constituting biofilms [133]. CLSM in combination with fluorescent probes can also be used to identify major biomolecules but it often results in nonspecific binding and misidentifi-cation [134]. Thus, the FISH technique allows identification, visualization, and quantifica-tion of specific bacteria within the biofilm microbial community. However, the procedure is complex and time-consuming. Another major disadvantage is that the dehydration step can destroy the original structure of bio-aggregates and biofilms.

EPS’, such as proteins and polysaccharides, identification and quantification can also be achieved with various methods. Sodium Dodecyl Sulphate–Polyacrylamide Gel Electrophoresis (SDS-PAGE) is an easy and low-cost electrophoresis method used to separate proteins, extracted from biofilms, by their molecular mass on a polyacrylamide gel, regardless of their charge due to SDS. This technique is often used to identify proteins in biofouled membrane bioreactors (MBRs) [129,130].

Matrix-Assisted Laser Desorption/Ionization-Time of Flight Mass Spectrometry (MALDI-TOF MS) is a new simple, fast and cost-effective tool for surface characterization. Proteins and peptides present in the biofilm are desorbed with a laser adsorbing solution (MALDI matrix) and analyzed in mass spectrometry (MS) to measure their exact size and signal intensity for quantification [24]. This technique also allows bacterial identification, as peptides and proteins are specific to each bacterial species [124].

EPS protein can also be quantified by the Bradford assay which is a colorimetric quantitative protein determination assay based on a shift of absorbance of a dye when binding with protein. A similar method can be used for polysaccharide content, where sugar groups are reduced by phenol and sulfuric acid and their absorbance is measured (DuBois assay) [135]. Sweity et al. used both this technique to study EPS adherence and viscoelastic properties in MBR [136]. They showed that fouling was linked to EPS adher-ence as a polysaccharide content increase resulted from a strong EPS adherence leading to a reduced membrane permeability.

**Figure 17:**
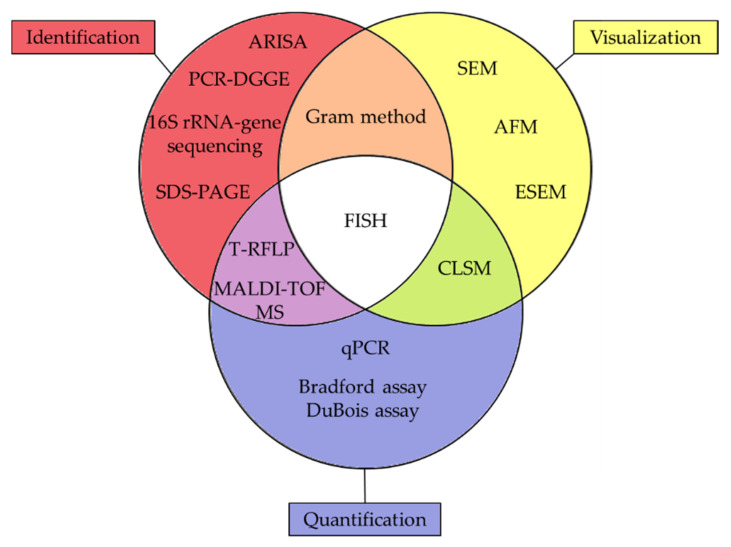
Overview of the different techniques presented to characterize biofilms on IEMs membranes.

All the techniques used to characterize membrane biofilm are shown in the summary Figure 17.

### 2.2. Characterization of the Interaction of Foulants with the Membrane Surface

#### 2.2.1. Localization of Foulants and Surface Roughness Parameters

As already mentioned in Section 2.1.1., SEM or SEM combined with EDS are widely used to determine the localization of mineral substances on the surface and in the volume of IEM [47,48,50,137]. Much less often, these methods are used to determine the localiza-tion of organic foulants [47] and to make comparisons with the surface roughness.

**Figure 18:**
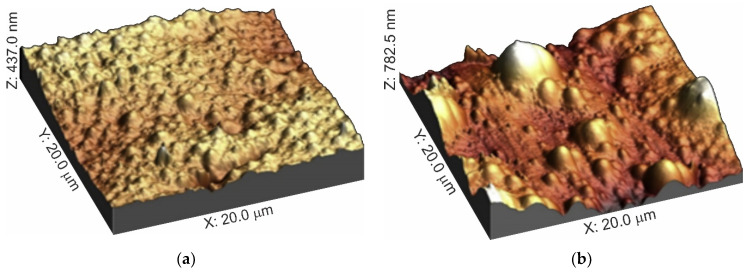
Three-dimensional atomic force microscopy AFM images representing surface of pristine (**a**) and fouled with peptides and amino acids (**b**) Neosepta AMX-Sb membrane. Adapted from [79].

Atomic force microscopy provides some insight into the presence of organic matter on membrane surfaces. Fouling with these substances (wine components, amino acids, proteins, etc.) leads to a change in adhesion forces [74,138,139], as well as surface rough-ness parameters in comparison with the pristine IEM [33,79,140]. Figure 18 shows an example of such changes in the case of membrane fouling during ED fractionation of pro-tein (snow crab by-products) hydrolysate [79]. It should be noted that the recorded param-eters of the fouled surface relief correspond to the operating conditions of IEM in real membrane stacks only when using an AFM (and SEM) modifications, which allow one to study swollen samples or membranes in water [141] because the size of organic foulants is highly dependent on the degree of hydration.

**Figure 19:**
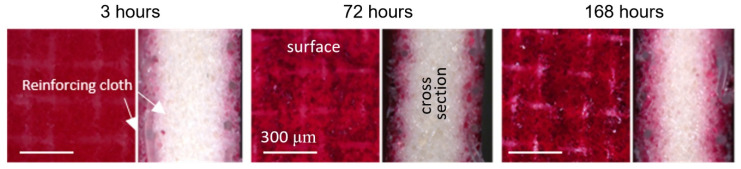
Changes in the color of the surface and cross-section of the MK-40 (Shchekinoazot LTD, Pervomaiskii, Russia) cation-exchange membrane during its fouling with anthocyanins (red color) and proanthocyanins (brownish-violet color), which are components of cranberry juice. The contact time of the membrane with cranberry juice in hours is indicated above the images.

Modern modifications of optical microscopy make it possible to examine the surface and cross-sections of swollen samples [33,67,73], to determine the localization of foulants (Figure 19) and the thickness of the film formed by them on the IEM surface, based on the difference in color of the pristine membrane and foulant.

**Figure 20:**
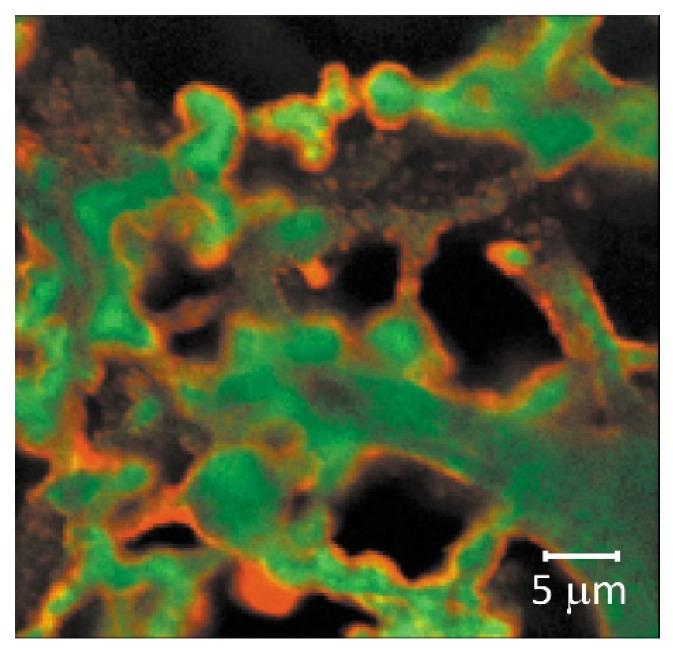
Confocal Laser Scanning Microscopy image of lysozyme protein adsorption on Sartobind S (Sartorius, Göttingen, Germany) cation exchange membrane in a 50 mM potassium phosphate buffer with pH = 7.5 at equilibrium. Membrane backbone was labelled with 6-DTAF (green) and the lysozyme was coupled to Cy3 dye (red) [139].

Confocal Laser Scanning Microscopy (CLSM) is a powerful technique to obtain sharp images of a sample that would otherwise appear blurred when viewed under a conven-tional microscope [97]. It uses the light reflected from the sample (epitransmission or re-flectance) or captures the fluorescent light that is excited in the sample by the incident beam (epifluorescence). One of the main advantages of this method is that potential or-ganic foulants, such as proteins, peptides, polysaccharides or biofoulants, could be stained with different fluorescent probes which facilitates the quantitative characterization and the visualization of fouling and polymer adsorption, and could lead to the de-termination of the possible interactions between proteins and membranes. For example, Reichert et al. [142] studied CLSM images to investigate the adsorption of two model proteins, BSA and lysozyme on commercially available CEM (Sartobind S) and AEM (Sartobind Q) for the protein purification process (Figure 20), Vaselbehagh et al. [143] com-pared CLSM images of PDA-modified AMX (Neosepta—Astom, Shunan, Japan) carried out to improve the biofouling resistance of the membrane during ED treatments and it was possible to clearly observe stained viable bacteria on the surface; Herzberg et al. identified living and dead cells on the surface of various CEMs and AEMs [144]. While interest in the CLSM method is growing, it remains less used than SEM and AFM.

**Figure 21:**
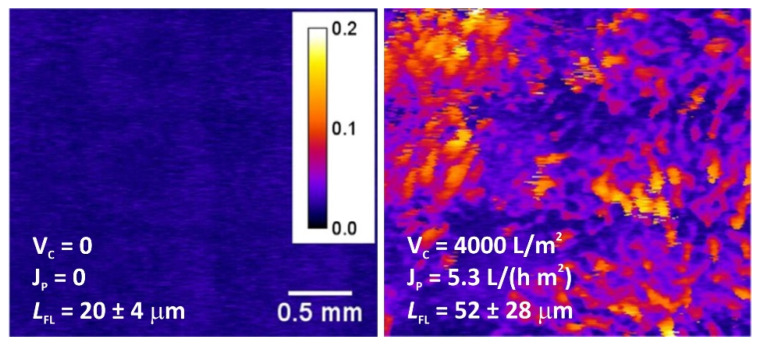
Optical Coherence Tomography 2D topographic image of the membrane/solution interface in distillation process depending on amount of produced condensate (referred to as cumulated volume V_C_.) and permeate flux, J_P_. The colored bar shows the height of the biofoulant layer above the surface of a flat membrane (in mm). $L_{FL}$ indicates the average height of the foulant layer. Adapted from [142].

Optical Coherence Tomography (OCT) enables in situ visualization and quantifica-tion of fouling layers dominated by scaling [145,146,147], monitoring of biofouling develop-ment in membrane [148], quantitative analysis of membrane fouling by oil emulsions [149], etc. An example of biofouling visualization obtained by this method is shown in Figure 21.

Another effective way to determine the thickness of the foulant film (and its electrical resistance) is to analyze the parameters of the high-frequency arc of the electrochemical impedance spectrum (EIS) obtained in the absence of a constant electric field [67,82,150,151,152,153]. For such analysis, mathematical models [154], as well as the method of electri-cal equivalent circuits [67,149,150] are used. If the foulant and the membrane material have significantly different values of electrical capacitance, an additional arc that charac-terizes the foulant layer appears in the high-frequency range of the impedance spectrum [67,147,149,150,151].

#### 2.2.2. Membrane Surface Charge and Hydrophobicity

Other techniques used to characterize the charge and degree of hydrophobicity of the membranes are very useful in determining if the fouling is impregnated or not. These techniques are global (several mm² of the surface) and ex-situ.

Contact angle. Organic foulants very often contain highly hydrated polar groups (carboxyl, hydroxyl, phosphoric acid, amino groups, etc.). Quite often these groups are attached to hydrophobic aromatic chains (polyphenols, aromatic amino acids, etc.). Therefore, the hydrophilic/hydrophobic balance of the IEM surface provides important information about foulants and the nature of their interaction with the IEM material. For example, fouling with phenylalanine (aromatic amino acids) [34], polyacrylamide [155] or bovine serum albumin and humic compounds [69] showed a significant increase in the surface hydrophobicity of fouled IEM as compared to the pristine one. In the case of phe-nolic compounds, the hydrophobicity decreases for AEMs while it always increases for CEMs [26,32,33] due to the different orientation of the hydrophilic and hydrophobic com-ponents of the foulant particles, which is determined by the sign of the electric charge of fixed membrane groups [31]. The hydrophilic/hydrophobic balance of a surface is usually determined by the values of the contact angle [26,31,32,33,34,69,152,156], which is formed between the tangent drawn to the surface of the liquid–gas phase and the IEM surface with the vertex located at the point of contact of the three phases. The contact angle is al-ways measured inside the liquid phase.

**Figure 22:**
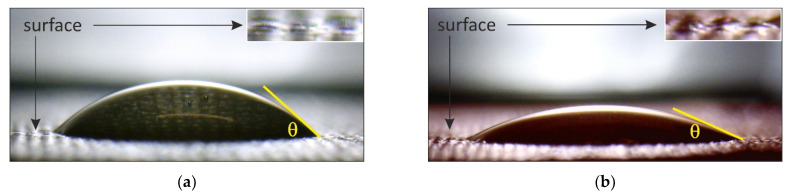
Contact angles of swollen CJMC-5 membrane (Hefei Chemjoy Polymer Material Co., Hefei, China) surface using sessile drop method: pristine (**a**) (soaked in 0.01 M NaCl solution, θ = 42 ± 10°) and fouled in red wine (**b**) (soaked for 3 days in a polyphenol extract from the wine pulp, θ = 24 ± 10°).

The sessile drop method is the most commonly used. In this method a drop of liquid is placed on the test surface (Figure 22). Despite the apparent simplicity of the method, the results depend on many factors [157,158,159]: the chemical nature and volume of the test liquid; the height from which the drop falls; the water content of the sample; the position of the sample relative to the gravitational field; the time elapsed since the drop touches the test surface; software used to process the obtained images; etc. That is why the data obtained by different researchers are often very different. Sometimes the error range turns out to be larger than that expected due to the changes in the hydrophilic/hydrophobic balance of the surface caused by fouling.

In addition, cracks can form on the IEM surface when dry samples are used in meas-urements. Contact of foulants with hydrophobic air can lead to a change in the orientation of their organic chains and, accordingly, to a change in the hydrophilic/hydrophobic bal-ance of the investigated surface in comparison with the aqueous medium. To break down these factors, contact angles are increasingly measured on swollen IEM samples [33,160]. The captive bubble method [161] also seems to be very promising but is used very rarely so far [162]. Note that all these techniques are relatively simple to implement.

Zeta (the electrokinetic) potential (ζ) is the electric potential measured at the fluid slipping plane along the IEM surface [163]. The slipping plane located at the boundary between the diffuse electric layer and the adsorption (dense) electric layer, or in the diffuse layer near this boundary. The diffuse layer contains mobile counterions, which are at-tracted to the IEM surface due to electrostatic forces. The adsorptive electric layer is directly adjacent to the membrane material and is formed as a result of electrostatic interactions of membrane fixed groups with counterions and specific adsorption of foulants. The magnitude of ζ can provide information about electrostatic or charge repulsion and attraction forces in the specific case of organic particles interactions with IEM [30,46,69,164,165,166] and plays a vital role in fouling. It is appropriate to measure this parameter before, during and after membrane operation in food industry processes [24]. However, only few studies have focused on ζ potential analysis for characterizing IEM fouling by proteins, peptides and amino acids or other organic foulants such as humate [114], since the current and standard automatic zeta potential measurement equipment is not suitable for IEMs. The point is that IEMs are conductors of the second kind (they participate in the ion transport), and this property significantly distorts the results.

Our view is that more reliable values of ζ, as well as surface charge, can be obtained from tangential streaming potential measurements. For example, a laboratory-made gap cell which described in detail by Sabbatovskii et al. [167] is applied in the paper [33]. This cell is similar to Anton Paar SurPASS 3 [168]. The streaming potential measurements are carried out using two Ag/AgCl electrodes connected to multimeter and an electrolyte solution pumped at the pressure drop between inlet and outlet of the channel in the range from 0.125 bar to 0.625 bar. The channel was formed by two identical IEMs.

**Figure 23:**
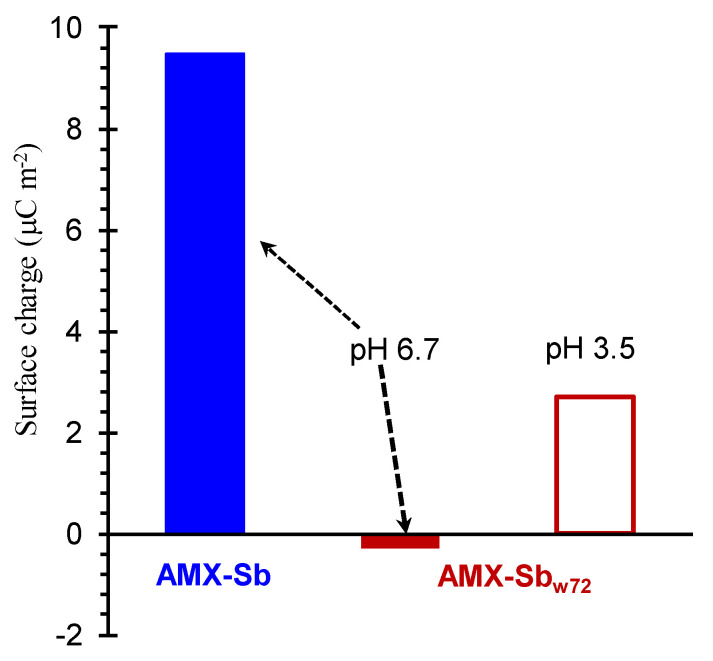
Surface charge of the pristine (AMX-Sb) and fouled in wine (AMX-Sb_w_) membranes in a 0.02 M NaCl solution with pH 6.7 and 3.5. Adapted from [33].

These studies showed that the surface charge of the anion-exchange membrane AMX-Sb contacted with wine changes from positive to negative when the pH of the feed solution changes from 3.5 to 6.7 (Figure 23). These data provided additional confirmation of anthocyanins (which change their electric charge depending on pH) adsorption by the IEM surface.

Note that the correct determination of the parameters of the hydrophilic/hydrophobic balance, as well as the zeta potential and surface charge, requires normalization to the true area of the investigated surface [169], which can be found, for example, using 3D SEM, AFM or profilometry data.

## 3. Mechanisms of Foulants Interaction with Ion-Exchange Materials

We should mention that one or more substances present in food, sometimes even in very low concentrations, with a high affinity for the membrane material, are usually re-sponsible for fouling. Some compounds can slowly adsorb on the surface and/or in the membrane bulk and irreversibly change its structure. Phenomenon of organic fouling of IEMs can be quick, cumulative and destructive at the same time during long term contact with the treated media. It depends on the membrane material and the fixed groups charge and the nature of interactions between organic particles and IEM. Let us examine the main mechanisms of foulants interaction with ion-exchange membranes.

### 3.1. Physicochemical Interactions

Electrostatic, hydrophobic–hydrophobic π–π (stacking) as well as ion–dipole (hy-drogen bonds) and dipole–dipole (Van der Waals) interactions underlie the fouling of ion-exchange materials.

Electrostatic interactions between counterions and IEM fixed groups are determined by the value of the membrane exchange capacity and the electric charge that the foulant has in solution (fouling of the IEM surface) or acquires upon penetration into the internal IEM solution (fouling of the IEM bulk). The vast majority of components that make up the liquid media of the food industry (amino acids, polybasic carboxylic and inorganic acids, proteins, anthocyanins, etc.) have amphoteric properties (they are ampholytes). They enter into protonation–deprotonation reactions with water and with each other. Therefore, their electric charge depends on the pH of the medium and the dissociation reaction (and protonation–deprotonation) constants of ampholytes (Ka). Proteins, which are components of milk, whey, and animal blood, exhibit the most complex behavior, because several amino acids with their own dissociation constants of amino groups and carboxyl groups are included in each of them.

**Figure 24:**
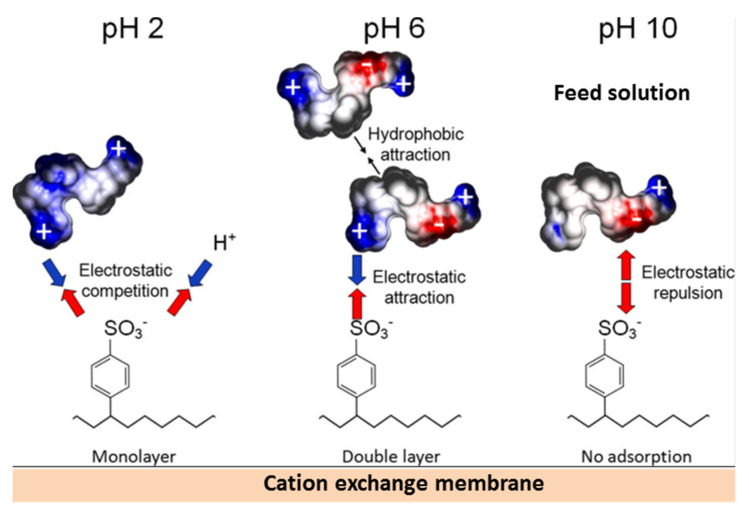
Schematic fouling mechanisms of CEM by peptides depending on their electrostatic charge and the pH of feeding whey solution. Adapted from [62].

Persico et al. [62,65,68] performed 3D mapping of the electrostatic charge of various whey proteins using molecular dynamics simulation and evaluated the effect of electro-static interactions on the possibility of IEM surface fouling with these high molecular weight substances. They found [62] that the interaction of positively charged fixed AEM groups with proteins that are enriched in carboxyl groups is most likely for the pH of the treated solution exceeding the pKa (≈4) of these groups. A decrease in quantity of amino groups in the composition of peptides will lead to the fact that their electrostatic interac-tions with AEM become more significant and more stable when the pH of the treated solu-tion exceeds the pKa of amino groups (≈10). The adsorption of a protein monolayer by the CEM surface is most probable in acidic solutions [63,68] (pH 2). In an alkaline solution (pH 10), the protein acquires a negative charge and is repelled from the similarly charged CEM surface (Figure 24).

The use of overlimiting current modes leads to alkalinization of the solution near the CEM surface and its acidification near the AEM surface due to water splitting [68]. The result of a shift in pH compared to bulk solution is the loss of electric charge by proteins or even the acquisition of a charge opposite to the charge of membrane fixed groups. These changes in the charge contribute to a reduction in IEM fouling with proteins. Low molec-ular weight amino acids and polybasic organic acid anions found in whey and other dairy products, as well as anthocyanins found in wine and juices, can enter the IEM. 

**Figure 25:**
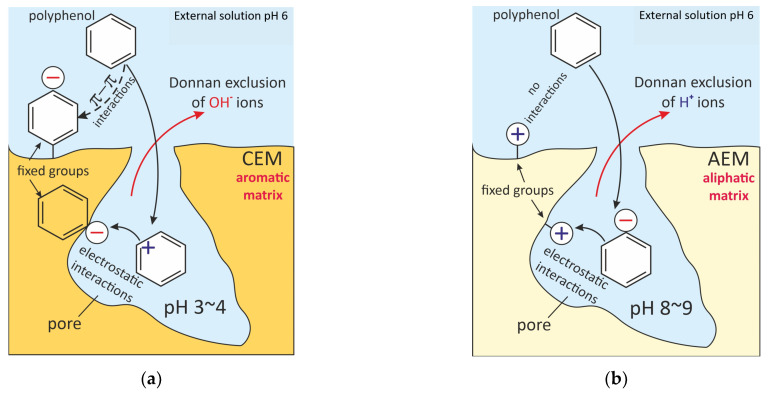
Schematic representation of the electrostatic interactions of polyphenols (anthocyanins) with aromatic CEMs (**a**) and aliphatic AEMs (**b**) in bathing solution with pH 6.

**Figure 26:**
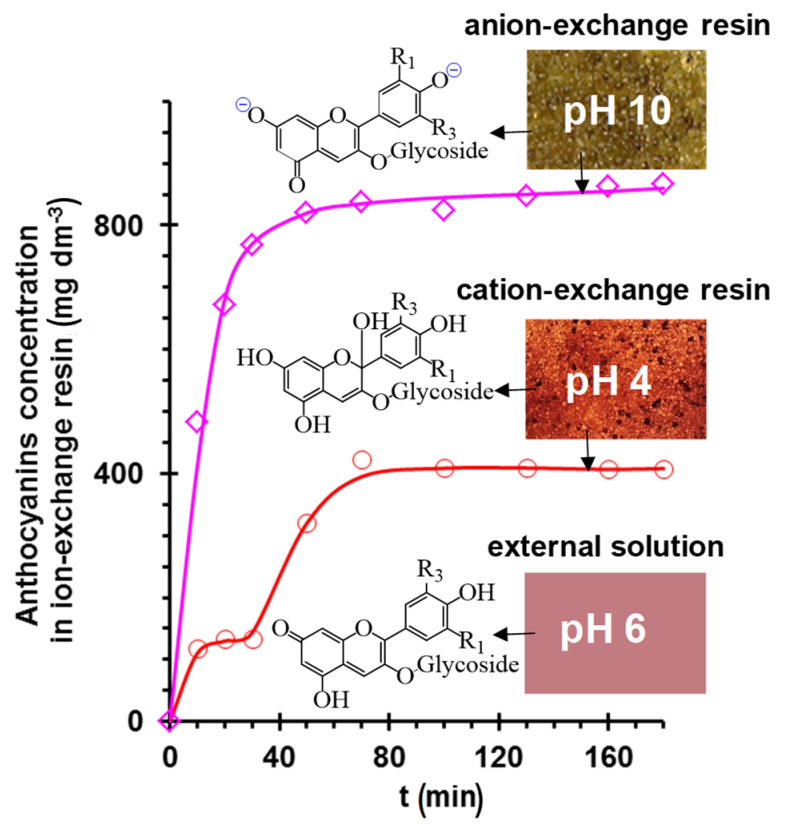
The anthocyanins adsorption by the ion-exchange resins vs time of it soaking in the aqueous solutions (pH 6) with anthocyanin concentration of 40 mg dm^−3^. The insets show the color of the external solution and the color of resins equilibrated with this solution, as well as the structures of anthocyanin species that correspond to these colors. Adapted from [92].

A schematic representation of the electrostatic interactions of polyphenols (anthocy-anins) with aromatic CEMs and aliphatic AEMs in bathing (external) solutions with pH 6 is shown in Figure 25. It has already been discussed in Section 2.1.2. that the pH of a CEM (or a cation exchange resins) internal solution is shifted to acidic values, while the pH of an AEM internal solution is shifted to alkaline values as compared to the external solution. The reason for this shift is the Donnan exclusion from ion exchange materials of hydroxyl ions (CEMs) or protons (AEMs), which are the products of water molecules and ampholytes protonation reactions [170,171,172,173]. The higher the membrane exchange capaci-ty, the stronger the Donnan effect [93]. The result of such a shift can be a change in the foulant electric charge inside the ion-exchange material as compared to the external solu-tion. Pismenskaya et al. [92,174] demonstrated this possibility using FTIR and color indi-cation of anthocyanin structure (Figure 12). For example, in an external solution with a pH of 6, anthocyanins have a guinoidal anhydrobase structure and have no electrical charge. At pH 4, which is established within an aromatic cation exchange resin (having negatively charged sulfonate groups), anthocyanins are mainly converted to carbinol pseudobase and hardly participate in electrostatic interactions. Inside the anion exchange resins, anthocyanins become singly charged (aromatic AV-17-8, LLC “CHIMIMPEX”, Moscow, Russia) or doubly charged (aliphatic EDE-10, PJSC “Uralchimplast”, Nizhny Tagil, Russia) anions and enter the electrostatic interactions with positively charged resin fixed groups. The result of this interaction is a higher sorption of anthocyanins by anion exchange resins than in the case of aromatic cation exchange resin (KU-2-8, LLC “CHIMIMPEX”, Moscow, Russia) at the pH 3 of the external solution (Figure 26). At the external solution pH = 3, on the contrary, anthocya-nins inside the anion exchange resins do not have an electric charge, but inside the cation exchange resin they are cations. As a result, the fouling of cation-exchange materials un-der the conditions of industrial ED processing of wines and juices (pH 3) is much higher than that observed for anion-exchange materials.

The influence of electrostatic interactions on the sorption of anthocyanins by ion-exchange materials, depending on their exchange capacity and the external solution pH, is also considered in [175,176,177,178,179,180]. Similar results were obtained in the case of IEM [31,33,73].

It should also be mentioned that some of the multiply charged counterions contained in liquid media of the food industry enter the electrostatic interactions not with one, but simultaneously with two fixed groups, causing an effect equivalent to additional cross-linking of the ion-exchange matrix [93]. Such interactions are typical, for example, for tri-ply charged anions of phosphoric acid and weakly basic groups of AEMs, as well as for doubly charged calcium anions and sulfonate groups of CEMs. It is known [93,181,182,183] that the latter enter the specific (donor–acceptor) interactions, which, in particular, are ex-pressed in the formation of weakly dissociating ion–ion associates “sulfo group - calcium ion”.

Hydrophobic–hydrophobic π–π (stacking) interactions generally occur if both the foulant and the IEM material contain aromatic rings. These interactions are often a key contributor to the fouling of IEMs and ion exchange resins in wine and juice processing. Indeed, even under conditions that are unfavorable for electrostatic interactions (Figure 26), the sorption of anthocyanins by the KU-2-8 resin is only two times less than in the case of the AV-17-8 resin, which has a similar aromatic matrix and structure. This sorp-tion is provided by π–π (stacking) interactions between the aromatic rings of polyphenols and the KU-2-8 polymer matrix. Similar results are presented in works [173,176,184,185], where an increase in the sorption of anthocyanins and other polyphenols is noted during the transition from an aliphatic to an aromatic polymer matrix of ion-exchange resins and membranes.

Two plateaus on kinetic (Figure 26) and equilibrium sorption isotherms and the re-sults of isotherm processing using the Freudlich model equation [186] allow us to con-clude that polymolecular adsorption of these substances [92] by ion exchange materials occurs due to the π–π (stacking) interaction of polyphenols with each other [75,187] or with other substances. Therefore, proanthocyanidins 2-mers, 3-mers, 4-mers and poly-mers are identified inside IEMs that were in contact with wine or cranberry juice [26,73]. These substances can probably form directly in membrane pores.

Table 2 summarizes ATR–FTIR data on CH-π and π–π interactions or hydrogen bonds formation between IEM materials and polyphenols that are contained in juice or wine.

Ion–dipole (hydrogen bonds) and dipole–dipole (Van der Waals) interactions are characteristic of foulants and IEMs which have oxygen-containing polar (hydroxyl, car-bonic, phosphonic, sulphonic, etc.) groups and hydrogen in fixed groups (primary and secondary amines) or aliphatic chains (materials such as polyamide, polytetrafluoroeth-ylene, polyvinyl chloride, etc.) [172,173,182]. According to [62], the hydrogen bonds and dipole–dipole interactions are the main reason for the formation of multilayered fouling on the CEM surface in protein-containing solutions at pH = 6 (Figure 24), that is, when there are no electrostatic interactions between proteins and fixed membrane groups. The aliphatic anion exchange resin EDE-10P sorbs 30% more polyphenols than the aromatic cation exchange resin KU-2-8 at an external solution pH = 3 [92], although anthocyanins inside EDE-10P are mostly in molecular form and are not able to enter the electrostatic in-teractions, while inside KU-2-8 anthocyanins are cations that interact with negatively charged fixed groups. The reason is the primary, secondary, and tertiary amines (which are fixed groups of the aliphatic resin) are able to form hydrogen bonds with hydroxyl groups of anthocyanins.

The ability of proteins and polyphenols (proanthocyanins and anthocyanins) to par-ticipate in almost all of the listed types of interactions with each other and with the IEM matrix predetermines the formation of colloidal particles in membrane pores and on their surface. It is known [71,193,194,195], for example, that polyphenols form high-molecular colloidal systems with carbonic acids, amino acids and saccharides, as well as with inorganic species, such as Ca^2+^ or Fe^3+^ [196] in wine and juices. Moreover, CEMs, which can contain Ca^2+^ or Fe^3+^ ions as counterions, have more favorable conditions for the formation and growth of colloidal particles inside pores (in situ) compared to AEM.

Perreault et al. [73] investigated the fouling of cation exchange membranes MK-40 (Shchekinoazot, Russia), CSE-fg (Astom, Shunan, Japan), CEM Type-II (Fujifilm, Tilburg, The Netherland) and CJMC-5 (Chemjoy Polymer Material Co. Ltd., Hefei, China) by polyphenols from cranberry juice (pH 2.45). They showed (according to the mechanisms described above) that CEMs with an aromatic matrix are more prone to fouling than CEMs with aliphatic matrix. Membranes with a lower exchange capacity lose it faster due to shielding of fixed groups by foulants. The thinner the membrane, and the more meso- and macropores it contains, the faster the fouling of its volume is completed. High molecular weight polyphenols mainly penetrate deep into the membrane through the macropores between the beads of the ion exchange resin and the inert binder, as well as through the extended macropores between the ion exchange material and the reinforcing cloth (if they take place) that reached the surface of the membrane.

### 3.2. Stretching of the Polymer ion Exchange Matrix

**Figure 27:**
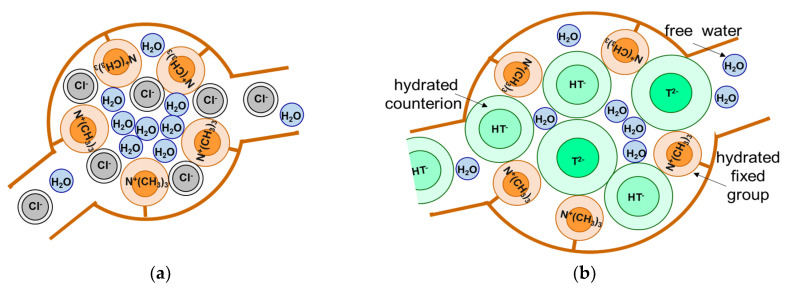
Scheme of free and bound water distribution and its effect on anion exchange membrane pore size in the case of weakly hydrated (**a**) or strongly hydrated (**b**) counterions: chlorides (Cl^−^) or hydrotartrates (HT^−^) and tartrates (T^2−^). Adapted from [171].

Typically, CEMs and AEMs after manufacturing are converted to Na^+^ or H^+^ and Cl^−^ or OH^−^ ionic forms, respectively. Before assembling of ED apparatuses, they swell in saline (NaCl) solutions. At the same time, most of the liquid media subjected to ED processing in the food industry contain highly hydrated anions of inorganic (carbonic, sulfuric, ortho-phosphoric, etc.) and organic (lactic, tartaric, malic, citric, etc.) acids, and also strongly hydrated cations, for example Ca^2+^, Mg^2+^ [197]. These ions displace less hydrated Cl^−^ and Na^+^ ions [194] in the pores of AEMs and CEMs. As a result, the fraction of bound water in the pores increases. Accordingly, the fraction of free water decreases in the internal IEM solution, which leads to an increase in osmotic pressure. This increase leads to the stretching of the elastic polymer matrix and, respectively, to increase in the membrane effective pore radius as compared to a state achieved upon contact of the IEM with less hydrated ions (Figure 27). The described phenomenon is well known [98] and has long been used to explain the change in the swelling of ion-exchange materials upon contact with an electrolyte and a solvent of various nature [198].

The influence of the components of the treated solutions on the elastic matrix state is of particular importance for explaining and predicting the consequences of fouling de-pending on the foulant nature. Therefore, in recent years, additional studies have been carried out [171] using the standard contact porosimetry method. It was found that the amount of bound water in the pores of AEMs actually increases if replacing NaCl solution with KHT, NaHCO_3_, NaH_2_PO_4_ solutions. In both homogeneous (AMX-Sb) and heteroge-neous (MA-41, Shchekinoazot, Russia; FTAM-EDE, FUMATECH BWT GmbH, Germany) membranes, the greatest increase in water content (and increase in size) is observed for micro- and mesopores with a radius from 1 to 13 μm. Swelling of ion-exchange material in heterogeneous membranes, apparently, leads to a decrease in adhesion between this material, the inert binder and the reinforcing material. This leads to an increase in the free water content and in the size of macropores in the places of contact between the particles of the ion-exchange resin and the inert binder, as well as between the ion-exchange com-posite and the threads of the reinforcing cloth.

The described phenomenon is the key for explaining the change in the IEM thickness, d, and their destruction during long-term (thousands of hours) operation in ED processing of the food industry liquid media. An increase in AEM thickness is observed, for example, in liquid media containing anions of polybasic carboxylic acids and/or phosphoric acid [171]. Moreover, in the case of a heterogeneous membrane MA-41, which contains a poly-styrene matrix regularly crosslinked with divinylbenzene, a new stable state of the matrix (characterized by the cessation of increase in d) is reached within ≈50 h after replacing the NaCl solution with solutions of polybasic acid anions. In the case of homogeneous membranes containing a randomly crosslinked copolymer of divinylbenzene and poly-styrene, an increase in d is more significant and continues abruptly throughout the observation period (more than 200 h). Apparently, the high osmotic pressure arising in the internal solution of such membranes leads not only to the stretching of the polymer matrix, but also to the rupture of the “bridges” that cross-link the polymer. The result is not only an increase in thickness, but also a gradual destruction of IEM, which is accelerated with an increase in the membrane operation time in ED processing of dairy products [157], as well as wine or juices [16,31,32]. These changes entail a loss of membrane mechanical strength [16,18,31,157]. For example, Garcia-Vasquez et al. [157] showed that the Young’s modulus (E), which can be related to the rigidity of a material, decreased by 20% for AEM (AMX-Sb, Astom, Shunan, Japan) at the end of their lifetime in a stack of ED used for whey demineralization. The breaking strength, which represents the membrane plasticity (break or/rupture point) decreased by 45%, and the area under the stress–strain curve decreased by almost 80%, which strongly indicated a loss of the material toughness.

**Figure 28:**
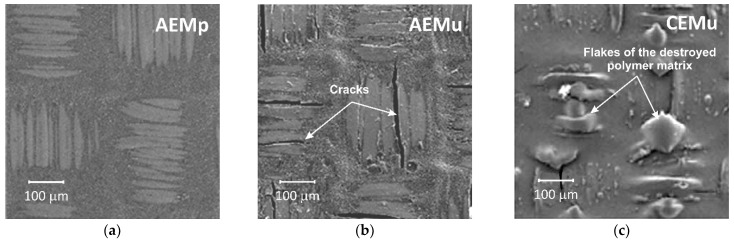
SEM images of the surface of AEMp pristine anion exchange membrane (**a**) as well as anion (AEMu) and cation (CEMu) exchange membranes used in industrial wine tartrate stabilization ED process (**b**,**c**) during 2500 and 2738 h, correspondently. Reconstructed from [31,32].

In the case of industrial ED processing of wines and juices, which is carried out at pH < 3.5, CEMs absorb more polyphenols (PP) than AEMs (see Section 3.1). The continuous growth of colloidal particles, which are compacted during the periodic cleaning of mem-brane stacks with chemical reagents, leads to a more significant stretching of the CEM ion exchange matrix [31,32]. As a result, with the same duration of operation in an ED appa-ratus, the cation-exchange membrane CMX-Sb (Astom, Shunan, Japan) is more destructed than the anion-exchange membrane AMX-Sb, which has a similar polymer matrix and reinforcing cloth (Figure 28).

Conversely, prolonged contact of the IEM with a foulant, which contains a small amount of bound water, can cause the ion exchange matrix to become denser. For exam-ple, Vasil’eva et al. observed a reduction in pore size, a decrease in thickness and flattening of the surface of profiled CEMs used for dialysis and electrodialysis processing of solu-tions containing the amino acid (phenylalanine) [199,200].

Thus, the rate and the degree of the ion-exchange matrix stretching (and destruction) are determined not only by the foulant nature, but also by the nature of the ion-exchange matrix (aliphatic, aromatic), as well as by the degree of its crosslinking [16]. In addition, as will be shown in Section 4, the cleaning conditions and the chemical nature of the rea-gents play an important role in this process [16,201,202]. The combination of these factors is necessary to predict the geometric parameters of CEMs and AEMs, as well as their me-chanical strength and susceptibility to degradation in the food industry ED processes.

Before concluding, we group the main techniques presented here and used to study the different aspects related to the fouling of ion exchange membranes in Table 3.

## 4. Conclusions

Electrodialysis is a very attractive method to use in the food industry. The attractive-ness of this method is primarily determined by the possibility of controlling the electrical charge of many substances (proteins, amino acids, particles of polybasic organic and in-organic acids, food dyes, etc.) by reagent-free pH control in the intermembrane space and within the IEM. This property provide unlimited possibilities in the separation and con-centration of valuable food and medicinal components, as well as in their purification from mineral impurities. The widespread use of ED in the food industry is constrained by the active interaction of the treated substances with ion-exchange membranes, which leads to a decrease in current efficiency, an increase in energy consumption and a de-crease in the life cycle of IEMs, which are the most expensive component of ED modules.

Recently, many new methods for studying fouling, as well as transport, mass trans-fer, and electrochemical characteristics of fouled membranes, have appeared. Their active use has led to a deeper understanding of interaction mechanisms of substances (constit-uent fluids of the food industry) with each other and with the IEM. It has been established that, in the case of ED application in the dairy industry and similar industries, the main reason for IEM fouling is electrostatic interactions of their fixed groups with proteins, as well as scaling of salts and hydroxides of alkaline earth metals (Ca^2+^, Mg^2+^, etc.).

In ED processing of liquid media of wineries, the tea industry and juice production, phenolic compounds (primarily anthocyanins) play a key role in fouling. Just like pro-teins, they can change their electrical charge depending on the pH of the environment. However, unlike the more massive proteins, anthocyanins can penetrate into the IEM and acquire an electrical charge that is different from their charge in solution. In addition, they actively enter the dipole–dipole (π–π, Van der Waals) interactions with each other and with the aromatic IEM matrix, which contributes to the formation of colloidal particles even in relatively small membrane pores. All IEM foulants in the food industry form hy-drogen bonds not only with fixed groups, but also with most of the polymers that make up membranes.

New knowledge has allowed broadening of our understanding of the reasons for the deterioration of IEM characteristics upon contact with liquid media of the food industry and more deliberate choosing of the appropriate ion exchange membranes and current modes. Thus, in the case of protein-containing solutions, this should be an IEM with a more hydrophobic surface in order to weaken the hydrogen bonding process. In the case of solutions containing phenolic compounds, it is better to use IEMs made from aliphatic materials, etc.

The main challenges for the near future are apparently the use of the accumulated knowledge (few π-bonds, low polar and more hydrophobic surfaces, a surface charge close to zero and minimal pH influence) to produce IEMs better adapted to the food industry (with the weakest protein-matrix and/or polyphenols–matrix interactions), or the selection of such membranes among the large number of IEMs that have recently appeared (Astom, Fujifilm, Mega (Auckland, New Zealand), Chimimpex (Wood Dale, IL, USA), HCPM (Wilmington, DE, USA), Du Pont (Wilmington, DE, USA), Hefei Chemjoy Polymer Materials Co. Ltd.). The production of these new IEMs can be achieved by searching for new material formulations (composites, co-polymers.) or by modifying the surfaces of existing IEMs. This last point is in itself a very vast domain.

## Figures and Tables

**Table 1:** Phenolic compounds identified in IEMs after tartaric stabilization of red wine by ED in industry (µg eq. Gallic acid/g dried membrane) during 6000 (CMX-Sb) and 1738 (AMX-Sb) hours. Adapted from [26].

Phenolic Compaunds	CMX-Sb	AMX-Sb
Quercetin	68.1 ± 9.6	40.4 ± 9.1
Quercetin-3-glucoside	10.9 ± 2.0	2.4 ± 0.9
Quercetin-3-galactoside	1.9 ± 1.1	3.3 ± 0.3
Quercetin-3-rhamnoside	-----	7.2 ±0.8
Kaempferol	2.0 ± 1.8	10.0 ± 0.8
Kaempferol-3-glucoside	2.6 ± 0.6	-----
Myricetin-3-glucoside	2.3 ± 1.4	-----
Isorhamnetin	176.8 ± 8.6	37.8 ± 8.9
4-hydroxylbenzoic acid	8.5 ± 3.3	44.3 ± 21.1
Protocatechic acid	28.0 ± 4.4	26.8 ± 2.8
Vanillic acid	9.1 ± 3.9	18.8 ± 5.5
Piceid	3.7 ± 2.9	1.4 ± 0.6
Resveratrol	5.5 ± 0.8	22.4 ± 4.7

**Table 2:** Assignment of characteristic bands of pristine and fouled CMX-Sb and AMX-Sb in the ATR–FTIR spectra and signs of foulant-matrix interactions [57].

	σ or Spectral Region (cm^−1^)	Characteristic Bands in Pristine IEMs	Indications of CH-π and π–π Interactions or Hydrogen Bonds in IEMs after ED Industrial Tartrate Stabilization of Wine
AMX-Sb	1200–1250 region Intense peak at 1240	N-C Stretching vibrations bands of functional sites [31,157,188]	Enlargement and intensification of bands by accumulation of phenolic acids [31] Appearance of –COOH band at 1168 cm^−1^ and C=O band at 1710 cm^−1^ [69]
	Doubled band at 1610 and 1625	N-H of functional sites [69,157]	
	1450, 1475, 1480, and 1510	Aromatic C=C stretching bands in aromatic ring of polystyrene (PS) [31]	Enlargement and intensification by accumulation of aromatic rings of phenolic compounds [41] Appearance of bands of the aromatic ring breathing modes and bands resulting from stretching and contracting of the C=C bonds in the range 1450–1615 cm^−1^ [157,189]
	Attached peaks at 2852, 2915 and 2967	Stretching vibrations bands of aliphatic –CH and –CH_2_– bonds in functionalized PS [16,18]	Enlargement and intensification of bands by accumulation of phenolic compounds in polymer matrix Blue-shift of bands to higher σ by 10 to 20 cm^−1^ under CH-π interactions [187]
	3010	Stretching vibrations of aromatic –CH– [190]	
	3400	Stretching vibrations band of –OH bonds [16]	Enlargement of the band by the appearance of a hydrogen-bonded region 3050–3350 cm^−1^ added to the non-hydrogen bonded band [16] Red-shift of the band to lower σ by 30 cm^−1^ under hydrogen bonds between linked water in polymer matrix and O of phenolic compounds [32,191]
CMX-Sb	1041	Stretching vibrations bands of S-O in –SO_3_^−^ sites [185]	Inhibition of –SO_3_^−^ functional sites by accumulation of colloidal particles of polyphenols or physical detachment of the sites from the polymer matrix by disruption of the sulfur-carbon bonds [32]
	1166	Stretching vibrations bands of SO_3_-H [192]	
	1186	Stretching vibrations bands of SO_3_-Na groups [159]	
	1485 and attached peaks at 1590 and 1650	Stretching bands of aromatic C=C bonds in aromatics rings of PS [16]	Intensification of bands by accumulation of phenolic compounds rich in aromatic rings and Peaks located at 1485 and 1590 cm^−1^ in new CMX are blue-shifted to higher wavenumbers by ~20 cm^−1^ under π-π and CH-π interactions [32,187]
	3400	Stretching vibrations band of –OH bonds [16,17]	Enlargement of the band by the appearance of a hydrogen-bonded region 3100–3300 cm^−1^ added to the non-hydrogen bonded band Red-shift of the band to lower σ by ~50 cm^−1^ under hydrogen bonds between linked water in polymer matrix and O of phenolic compounds [32]

**Table 3:** Summary table of the different techniques used to study the various aspects related to the fouling of ion-exchange membranes.

Technique	Application	Device Complexity	Interpretation Complexity	Use Frequency
	I: Identification V: Visualisation Q: Quantification	H: High M: Middle L: Low		
2D fluorescence/Fourier transform infrared correlation spectroscopy	V, I	H	H	L
31P nuclear magnetic resonance spectroscopy	I	H	M	L
Atomic force microscopy (AFM)		H	L	M
Classical optical microscopy	V	L	M	H
Combined with energy dispersive X-ray spectrometry (EDS)	I	H	H	L
Confocal laser scanning microscopy (CLSM)	V	H	M	L
Contact angle	Q	M	L	H
Fluorescence excitation-emission matrix (EEM)	Q, I	H	H	L
Fluorescence spectroscopy	I	H	M	L
Fourier transform-ion cyclotron resonance-mass spectrometry (FT-ICR-MS)	I	H	H	L
High-liquid performance chromatography (HPLC)	Q	M	L	M
High-resolution optical microscopy	V	M	M	M
Inductively coupled plasma optical emission spectrometry	I	M	H	L
Mass spectrometry (MS) coupled	I	H	M	L
Molybdate colorimetry inductively coupled plasma optical emission	I	M	M	L
Optical coherence tomography (OCT)	I	H	M	L
Optical microscopy combined with a color scale for pH indication	V	L	L	L
Raman spectroscopy	I	H	M	M
Reflectance–Fourier-transform infrared (ATR–FTIR)	I	M	M	H
Rutherford backscattering spectroscopy (RBS)	I	H	H	L
Scanning electrochemical microscopy (SECM)	V	M	L	M
Scanning electron microscopy (SEM)	V	H	L	H
Scanning ion conductance microscopy (SICM)	V	H	L	L
Size-exclusion (SEC)	I	M	L	M
Smear-prints	V	M	M	L
Sodium dodecyl sulphate–polyacrylamide gel electrophoresis (SDS-PAGE)	I	M	M	L
Standard contact porosimetry method	Q	L	M	L
Surface plasmon resonance (SPR)	I	H	H	L
Surface-enhanced Raman spectroscopy (SERS)	V, I	H	H	L
Synchrotron Fourier transform infrared mapping	V, I	H	H	L
Tip-enhanced Raman spectroscopy (TERS)	V, I	H	H	L
Total nitrogen content analysis Dumas method	Q	M	M	L
Total nitrogen content analysis LECO nitrogen quantification	Q	M	M	L
Ultra-high-liquid performance chromatography (UPLC)	Q	M	L	L
X-ray absorption fine structure (EXAFS)	I	H	M	L
X-ray diffraction (XRD)	I	H	L	L
X-ray photoelectron spectroscopy (XPS)	V, I	H	M	M
Zeta (the electrokinetic) potential	Q	H	M	L

## Abbreviations

All other abbreviations have their usual meaning, or are sufficiently explained in the text.

AEM	Anion-exchange membrane
AFM	Atomic force microscopy
ARISA	Automated ribosomal intergenic spacer analysis
ATR–FTIR	Reflectance–Fourier-transform infrared spectroscopy
BSA	Bovine serum albumin
CEM	Cation-exchange membrane
CLSM	Confocal laser scanning microscopy
ED	Electrodialysis
EDS	Energy dispersive X-ray spectrometry
EEM	Excitation-emission matrix
EIS	Electrochemical impedance spectroscopy (spectrum)
EXAFS	X-ray absorption fine structure
FISH	Fluorescence in situ hybridization
FT-ICR-MS	Fourier transform-ion cyclotron resonance-mass spectrometry
HPLC	High-liquid performance chromatography
HPLC-MS	Mass spectrometry coupled to the HPLC
IEC	Ion exchange capacity
IEM	Ion-exchange membrane
MS	Mass spectrometry
MW	Moleculer weight
OCT	Optical coherence tomography
PARAFAC	Parallel factor analysis
PCR-DGGE	Polymerase chain reaction denaturing gradient gel electrophoresis
PDA	Polydopamine
PEF	Pulsed electric field
PP	Polyphenol
PS	Polystyrene
PVDF	Polyvinylidene fluoride
RBS	Rutherford backscattering spectroscopy
RO	Reverse osmosis
SDS	Sodium dodecyl sulphate
SDS-PAGE	Sodium dodecyl sulphate–polyacrylamide gel electrophoresis
SEC	Size-exclusion chromatography
SECM	Scanning electrochemical microscopy
SEM	Scanning electron microscopy
SERS	Surface-enhanced Raman spectroscopy
SICM	Scanning ion conductance microscopy
SPHS	Soy protein hydrolysate solution
SPR	Surface plasmon resonance
TERS	Tip-enhanced Raman spectroscopy
T-RFLP	Terminal restriction fragment length polymorphism
UF	Ultrafiltration
UPLC	Ultra-high-liquid performance chromatography
UPLC-MS	Mass spectrometry coupled to the UPLC
XPS	X-ray photoelectron spectroscopy
XRD	X-ray diffraction
